# *In vitro* elucidation of antioxidant, antiproliferative, and apoptotic potential of yeast-derived β-1,3-glucan particles against cervical cancer cells

**DOI:** 10.3389/fonc.2022.942075

**Published:** 2022-08-18

**Authors:** Tarun Kumar Upadhyay, Rashmi Trivedi, Fahad Khan, Lamya Ahmed Al-Keridis, Pratibha Pandey, Amit Baran Sharangi, Nawaf Alshammari, Nadiya M. Abdullah, Dharmendra Kumar Yadav, Mohd Saeed

**Affiliations:** ^1^ Department of Biotechnology, Parul Institute of Applied Sciences and Centre of Research for Development, Parul University, Vadodara, India; ^2^ Department of Biotechnology, Noida Institute of Engineering and Technology, Greater Noida, India; ^3^ Biology Department, Faculty of Science, Princess Nourah Bint Abdulrahman University, Riyadh, Saudi Arabia; ^4^ Department of Plantation, Spices, Medicinal and Aromatic Crops, Bidhan Chandra Krishi Viswavidyalaya (BCKV)-Agricultural University, Mohanpur, India; ^5^ Department of Biology, College of Sciences, University of Hail, Hail, Saudi Arabia; ^6^ Department of Biology, College of Science, Imam Abdulrahman Bin Faisal University, Dammam, Saudi Arabia; ^7^ Department of Pharmacy and Gachon Institute of Pharmaceutical Science, College of Pharmacy, Gachon University, Yeonsu-gu, Incheon, South Korea

**Keywords:** β-glucan, antioxidant activity, apoptosis, anticancer, cervical cancer, ROS generation

## Abstract

Cancer is the leading cause of mortality worldwide and in particular is the fourth most common cause of mortality in women every year. Conventional treatments for cancer are chemotherapy and radiation therapy, which have various kinds of side effects. Hence, there is a high need to develop alternative, efficient, and safer therapies for cancer treatment. β-Glucan, a novel polysaccharide isolated from baker’s yeast *Saccharomyces cerevisiae*, shows noteworthy cytotoxicity toward a variety of cancer cell lines *in vitro*. In this research, we characterized the β-glucan with high-performance thin-layer chromatography (HPTLC) analysis and found that d-glucose units with β-1,3 links are the major component of the extracted β-glucan particles. Fourier transform IR (FTIR) analysis confirmed a β-(1→3)-linked glucan structure. *In vitro* cell cytotoxicity was evaluated by MTT with IC_50_ 136 μg/ml, and therapeutic potential was assessed by various assays using values below and above the IC_50_. A significant reactive oxygen species (ROS) generation at 50–150 μg/ml of concentrations indicated the apoptosis of cervical cancer cells. Along with ROS generation, these concentrations were also found to induce morphological changes such as fragmentation in DNA upon staining HeLa cells with DAPI. Mitochondrial membrane potential was significantly reduced after increasing the dose of treatment, assessed with the help of MitoTracker dye. Hence, by all these experimental supports, we observed that β-glucan has the potential to slow down the growth of cervical cancer cells, and it can be further investigated for unfolding its complete anticancer potential.

## Introduction

The most frequent malignant tumor of the cervix is cervical cancer, and it is the fourth leading cause of women’s death worldwide annually. It is a critical effect of infection with human papillomavirus, which can infect the cervical mucosa (1, 2). Cervical cancer caused 604,000 new cases and 342,000 deaths as reported by the World Health Organization in 2020 (3). It occurs most frequently in less-developed countries due to less availability of infrastructure and resources and limited treatment programs. Interestingly, cervical cancer develops at a very slow pace in the beginning and has no symptoms until it progresses to the point where it is fatal. Screening at the precancerous stage can help prevent the invasion of cancer (4). Once it becomes invasive, the treatment for cervical cancer also becomes costly and more extensive. Apart from being costly, cervical cancer treatments including chemotherapy and radiation therapy have many side effects. Hence, there is a need to develop therapies with higher efficiency and lesser side effects. Naturally occurring polysaccharides are used as therapies for various diseases and new research, and advancements in these therapeutics could pave the way for effective cancer treatment.

β-Glucan is a polymer of glucose monomers in different forms. It can have different properties such as alpha–beta isomeric form and differences in solubility, branching, and chain length. These properties are essential for β-glucan to become effective and do their function properly (5). In many studies, it is found that β-glucan can boost neutrophils and macrophages by causing them to produce pro-inflammatory cytokines and chemokines, resulting in an oxidative burst (6).

β-Glucan has a rigid structure having β-(1,3 and 1,6) glycosidic linkages in the case of mushrooms and yeast while having β-(1,3 and 1,4) glycosidic linkage in cereals (7). There are several parameters responsible for the biological activity of β-glucan like molecular weight, structure, the density of branching, charge on polymer, and their conformation on whether it is a single helix, triple helix, or random coil (8, 9). Any change or modifications in β-glucan conformation can affect the function and immunomodulatory properties of β-glucan. β-Glucan, being a polysaccharide that is insoluble in water, is potentially used in the food and pharmaceutical industry (10), which works as raw materials for various kinds of beverages and medicines and helps in the treatment of many diseases like cancer, diabetes, and high cholesterol levels as well. It is shown by many studies that β-glucan derived from yeast has the potential to activate macrophage cells to initiate many functions like lipid-lowering activity (11), antibacterial activity (12), and antioxidant activity (13), including anticancer activity (14).

Zymosan is an insoluble form of yeast-derived β-glucan that increases the number and activity of macrophages along with activating the complement system (15). β-Glucan binds with the transmembrane receptor Dectin-1 and regulates the immune responses (16–18). In a clinical trial, oral β-glucan in advanced breast cancer patients stimulated the proliferation and activation of monocytes (19). Moreover, fungal β-glucan was found to manipulate the innate and adaptive immune systems leading to the effective manipulation of the tumor microenvironment (20). Maitake D-Fraction containing β-glucan from Maitake mushroom was able to decrease the tumor size in breast, liver, and lung cancers (21).

β-Glucan has an immunomodulatory role and can activate T cells, natural killer cells, macrophages, and complement pathways leading to the modulation of very important signaling pathways that are involved in the apoptosis of cells. Many studies have evaluated the role of β-glucan on cancer cells and suggested that β-glucan can work as a potent immunomodulator and anticancer agent. In this study, we focused on exploring the anticancer potential of β-glucan as one of the alternative efficient and safer therapies against cervical cancer cells with the help of various assays.

## Materials and method

### Reagents and chemicals

Anthrone reagent (Thermo Scientific, Waltham, MA, USA), sulfuric acid (HiMedia, Mumbai, India), Congo red (HiMedia), *n*-butyl alcohol (HiMedia), methanol (HiMedia), hematoxylin (Mayer’s reagent) (HiMedia), acetic anhydride (Sigma-Aldrich, St. Louis, MO, USA), chloroform (HiMedia), ammonium buffer solution (HiMedia), hydrochloric acid (HiMedia), lead(II) acetate trihydrate (HiMedia), ninhydrin (HiMedia), Fehling A and B solutions (HiMedia), potassium ferricyanide (HiMedia), trichloroacetic acid (HiMedia), DPPH (2,2-diphenyl-1-picrylhydrazyl) (Sigma-Aldrich), 3-(4,5-dimethylthiazol-2-yl)-2,5-diphenyl tetrazolium bromide (MTT) (Invitrogen, Carlsbad, CA, USA), (H2DCFDA) (dichlorofluorescein diacetate) (Invitrogen), LysoTracker Red (Invitrogen), and MitoTracker Red CMX-ROS (Invitrogen).

### Cell culture and maintenance

Cervical cancer cell line HeLa was procured from National Centre for Cell Science (NCCS, Pune, India). All the cell culture products including Dulbecco’s modified Eagle medium (DMEM), fetal bovine serum (FBS), antibiotic, and antimycotic solution were purchased from Gibco™ (Thermo Fisher Scientific). Cells were maintained in a humidified environment at 37°C and 5% CO_2_. Cell culture media DMEM was supplemented with 10% FBS and 1% antibiotic and antimycotic solution.

### Extraction procedure of β-glucan from baker’s yeast *Saccharomyces cerevisiae*

β-Glucan from baker’s yeast *Saccharomyces cerevisiae* was extracted according to the previously described protocol. In brief, dry yeast *S. cerevisiae* was purchased from the local market of Vadodara, Gujarat. Dry baker’s yeast measuring 40 g was suspended in 400 ml of 1 M NaOH and continuously stirred at 60°C for 30 min on a magnetic stirrer. The resulting material was kept in a water bath and heated at 80°C for 60 min. Further centrifugation was done at 9,000 rpm for 10 min, and obtained sediment was washed with triple distilled water (TDW). After sediment washing, pH was adjusted to 4–5 with the help of hydrochloric acid and incubated at 55°C for 60 min. After incubation, the alkali-insoluble solid material was collected and washed with TDW. Semisolid glucan particles were sonicated for 5 min. Homogenization was carried out at 1,000 rpm for 10 min, and finally, the suspension was dried with the help of a Labultima spray dryer. The structure of yielded β-glucan was elaborated with Fourier transform IR (FTIR) analysis, and an anthrone test was carried out for the confirmation of the nature of the carbohydrate (22).

### Anthrone test

Anthrone test was performed to confirm the presence of carbohydrates, with slight modifications in the previously described protocol. Briefly, 0.1 g of anthrone reagent was mixed with concentrated sulfuric acid. Stock solution measuring 25 ml was made by dissolving 1 mg/ml of β-glucan in distilled water. Carbohydrate standards were prepared in the range of 200–1,000 μg/ml with the appropriate dilution. Further, 4 ml of anthrone–sulfuric acid solution was added to all the samples, and the solution was incubated at 90°C for 30 min. After incubation, the solution was cooled up to room temperature, and absorbance was measured at 625 nm with Kanad Vidyut 8mpc Plus Clinical Biochemistry Analyzer (23).

### Fourier transform IR analysis

FTIR spectroscopy is for the structural analysis of polysaccharides on the position and anomeric configuration of glycosidic linkages in β-glucan (24, 25). FTIR spectra were obtained with a Bruker alpha FTIR spectrophotometer instrument using 16 scans from 4,000 to 500 cm^−1^.

### Congo red staining

Congo red staining is a colorimetric assay used to determine the interactions between Congo red dye and β-glucan. In our study, we evaluated the interaction by the previously described protocol with the help of a bathochromic shift (26). Congo red mixture measuring 163 μM was made by dissolving in phosphate-buffered saline (PBS) (at pH 7.2). Equal amounts of β-glucan and Congo red are mixed, and absorption spectra were recorded with a UV–vis spectrophotometer (Shimadzu, Kyoto, Japan; UV1900).

### High-performance thin-layer chromatography analysis

High-performance thin-layer chromatography (HPTLC) analysis was performed according to the previously described protocol (27). Yeast β-glucan dissolved in 90% HPLC-grade methanol, 2 μl of a 5 mg/ml solution of d-glucose and β-glucan, and 2 μl of a 10 mg/ml solution of d-glucose and β-glucan were applied on a 100 × 100 mm silica gel plate (Merck, Darmstadt, Germany). The bands were 8 mm wide with the first position at 15.0 mm and a distance of 13.4 mm. *N*-Butanol/methanol/water was used for the separation in a ratio of 5.2:2.7:2.1 (v/v/v). Images were visualized in UV at 254 nm.

### Size analysis

The size of the β-glucan particles was measured by Malvern Zeta sizer Ver. 7.03. Zeta sizer measures the size by the procedure of dynamic light scattering. Zeta sizer uses laser light to illuminate the particle and analyzes the fluctuation in intensity of scattered light. It measures the Brownian motion and correlates it with the particle size (28).

### Morphological analysis by scanning electron microscopy of β-glucan

Morphological analysis of the particle surface was performed for the observation of the size and shape of the particles (29). Particle morphology was visualized by scanning electron microscope (Hitachi S-3400) at an accelerating voltage of 15,000 V at different magnifications.

### Reducing power assay

The reducing power of the β-glucan was assessed by the previously described protocol (30). In brief, β-glucan sample in concentrations of 50, 100, 150, 200, 250, 300, 350, 400, 450, and 500 μg/ml in distilled water, (1% w/v) potassium ferricyanide, and (pH 6.6) PBS (2.5 ml each) were mixed, and the mixture was incubated at 50°C in the water bath for 20 min. Further, 2.5 of 10% trichloroacetic acid (w/v) was added, and the mixture was centrifuged at 2,000 rpm for 5 min. Obtained supernatant measuring 2 ml was mixed with 2 ml of distilled water and ferric chloride (0.4 ml, 0.1%, w/v), and absorbance was measured at 700 nm with a UV–vis spectrophotometer (Shimadzu UV1900).

### DPPH radical scavenging activity

Radical scavenging activity is the ability of a compound to reduce the 2,2-diphenyl-1-picrylhydrazyl radical (DPPH, Sigma). A radical scavenging activity test was performed according to the previously described method, with slight modifications (31). DPPH solution was made by dissolving DPPH in 95% methanol. Further, 0.2 mM of DPPH solution (4 ml) was added to 20, 40, 60, 80, and 100 μg/ml of concentrations of yeast β-glucan. The mixture was incubated at room temperature in the dark for 20 min. Absorbance was measured at 517 nm using a UV–vis spectrophotometer. % DPPH radical scavenging activity was calculated as follows:


$$\%\text{RSA}=\frac{\text{Absorbance of control}-\text{Absorbance of sample}}{\text{Absorbance of control}}\times 100$$

where control was taken as DPPH dissolved in methanol.

### Antiproliferative assay

Cell viability of the HeLa cells was assessed with the help of the previously described protocol (32). Briefly, 1 × 10^4^ cells were seeded in 96-well plates and treated with 50, 100, 150, and 200 μg/ml doses of β-glucan to make a final volume of 200 μl in each well for 24 h. After incubation, 10 µl of MTT dye (5 mg/ml) was added to each well and incubated for 4 h in dark. After that, 100 µl of dimethyl sulfoxide (DMSO) was added to dissolve the purple-colored formazan crystals, and absorbance was taken using a Readwell Robonik Elisa Reader at 570 nm.

### Reactive oxygen species generation assay

Reactive oxygen species (ROS) generation in the cervical cancer cells (HeLa) was measured according to the previously described protocol (33); 1 × 10^5^ HeLa cells/well were seeded in a 12-well plate in DMEM overnight at 37°C. After 24 h of incubation, cells were treated with 50, 100, and 150 μg/ml doses of β-glucan and again kept for 24-h incubation. After the incubation period was over, H2DCFDA dye was used to monitor cellular ROS generation. Cells were incubated with H2DCFDA (10 μM) for 15 min, and microscopy images of ROS generation were obtained.

### DAPI staining assay

DAPI staining assay was performed to observe the morphology of the apoptotic cells; 1 × 10^5^ cells/well were seeded in 12-well plates and treated with 50, 100, and 150 μg/ml doses of β-glucan for 24 h. After incubation, treated cells were stained with 1 μg/ml of DAPI (4′,6-diamidino-2-phenylindole) for 15 min. Morphological changes were observed and photographed (34).

### LysoTracker staining

LysoTracker Red staining was performed for detection of the acidic cellular compartments, according to the previously described protocol (35), with minor modifications. Briefly, 1 × 10^5^ cells/well were seeded in 12-well plates and treated with 50, 100, and 150 μg/ml doses of β-glucan for 24 h. After incubation, treated cells were stained with a 50 nM concentration of LysoTracker Red (Invitrogen) and imaged, and microscopy images were taken after incubation of 30 min.

### MitoTracker Red CMX-ROS staining

Measurement of mitochondrial membrane potential was carried out with the help of MitoTracker Red CMX-ROS according to the previously described protocol, with slight modifications. In brief, 1 × 10^5^ cells/well were seeded in 12-well plates and treated with 50, 100, and 150 μg/ml doses of β-glucan for 24 h. After incubation, treated cells were stained with a 200 nM concentration of MitoTracker Red CMX-ROS (Invitrogen) (36). After staining, microscopy images were obtained.

### Statistical analysis

All of the cell culture experiments were performed in triplicates. One-way ANOVA was applied using GraphPad Prism 8.0. A probability value of p < 0.05 was considered to be statistically significant. All the data were expressed as the mean ± standard deviation of the mean (SD) from triplicate experiments.

## Results

### Extraction of β-glucan from baker’s yeast *Saccharomyces cerevisiae*

**Figure 1:**
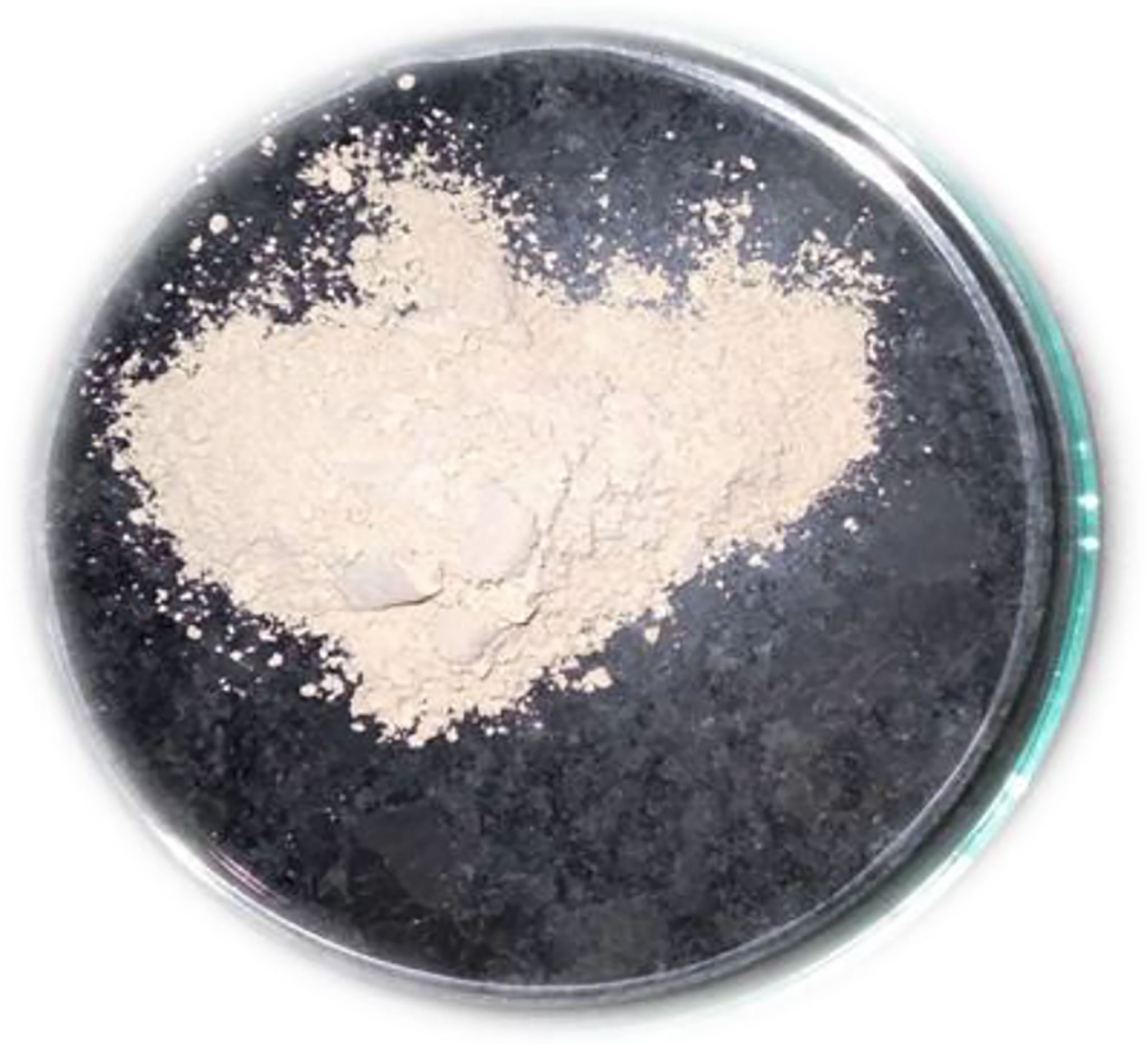
Extracted and spray-dried β-glucan powder from baker’s yeast *Saccharomyces cerevisiae*.

From 40 g of yeast, a total of 2.24 g of glucan particles was obtained after spray drying. Spray-dried particles were in white powder form as shown in **Figure 1**. The total yield obtained from 40 g of yeast was 5.6%.

### Anthrone test for quantitative estimation of carbohydrate

**Figure 2:**
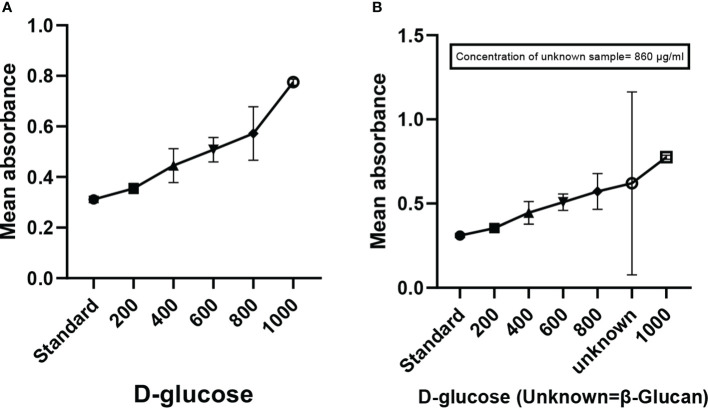
Anthrone test showing the quantification of carbohydrates (β-glucan). **(A)** Graph showing the increase in absorbance with the increase in concentration of sugar. **(B)** Graph of quantitative estimation of unknown sample. All the data expressed as the mean ± standard deviation of the mean (SD) from triplicate experiments.

Mixing of yellow-colored powdered anthrone reagent in sulfuric acid resulted in a yellow color solution. The addition of this solution to the sugar samples in the ranges of (200 to 1,000 μg/ml) as shown in **Figure 2A** and the β-glucan sample (unknown concentration) gave a blue-green color complex furfural. This furfural further reacted with anthrone to form a bluish-green color complex, which was determined colorimetrically. The concentration of the unknown sample in the quantitative estimation test was found to be 860 μg/ml (**Figure 2B**). The calibration curve showed a p < 0.0001 and an R^2^ value of 0.9201, indicating highly significant levels of obtained results. The error bars of the graph show the reproducibility of the assay.

### Fourier transform IR analysis

**Figure 3:**
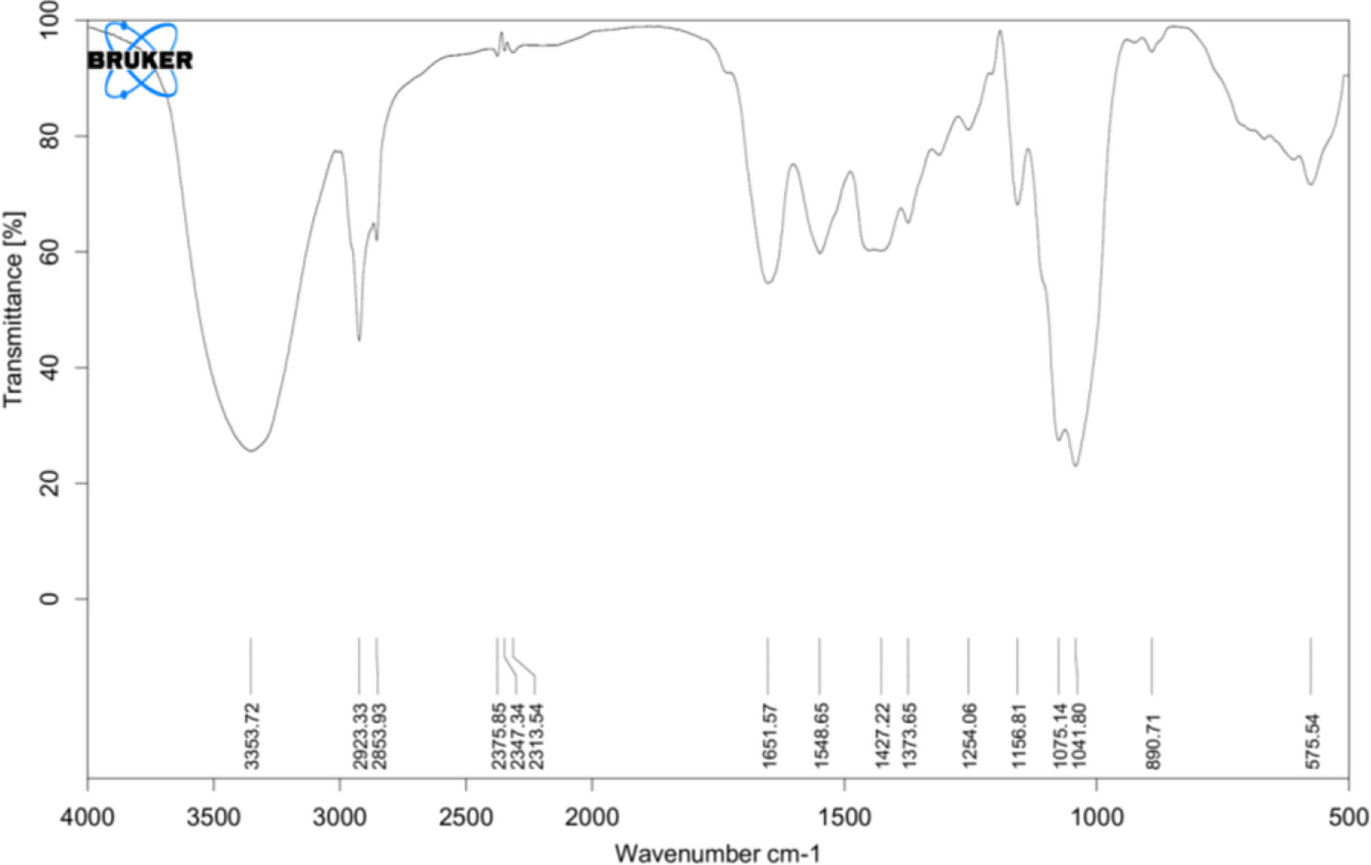
FTIR analysis: transmittance spectra of yeast β-glucan confirming the nature of polysaccharides and showing the presence of 1,3 as a major linkage in β-glucan sample. FTIR, Fourier transform IR.

FTIR spectra were recorded and compared with the yeast β-glucan spectra observed by Boutros et al. (2022) (37). FTIR analysis spectra were recorded between 4,000 and 500 cm^−1^, and % transmittance was observed. In spectra defined by Boutros et al. (2022), β-glucan band at a wavenumber of 890 was for beta-linked polymer, 1,076 for C–O stretch, 1,372 for CHOH stretch, and 2,919 for C–H stretch, and 3,390 was indicative of OH stretch. A similar pattern with a slight difference was observed in our analysis of yeast β-glucan transmittance spectra (**Figure 3**). Peak at wavenumber 890 showed beta-linked polymer, and 1,075 confirmed the presence of C–O stretch. The peak at wavenumber 1,373 indicated the presence of CHOH, and 2,923 indicated the presence of C–H stretch. OH stretch was found at 3,358 in our study. All these peaks were indicative of the 1,3 as the major linkage present in the β-glucan sample.

### Congo red staining

**Figure 4:**
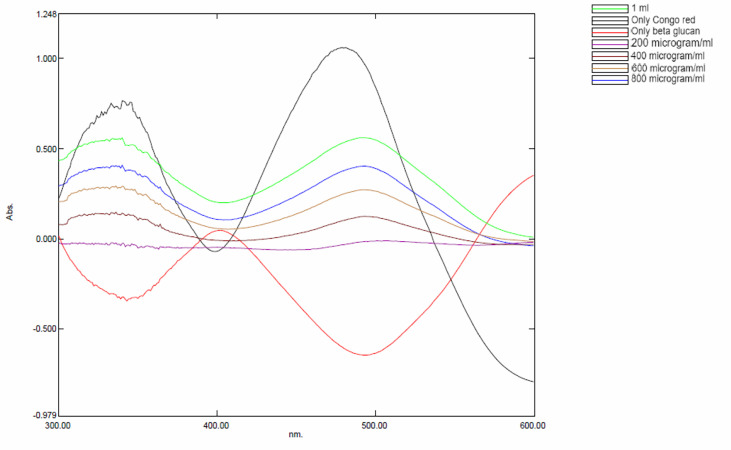
Bathochromic shift observed in yeast β-glucan and Congo red mixture.

Specific interactions between yeast β-glucan and Congo red dye were detected by the Congo red staining assay. In the visible absorption maximum of Congo red, a bathochromic shift from 300 to 600 nm was observed. Absorption spectra from 300 to 600 nm were recorded for a mixture of Congo red and yeast β-glucan in the range of 200–1,000 μg/ml. All the obtained spectra showed a bathochromic shift as shown in **Figure 4** and were in the agreement with previously published work by (26).

### High-performance thin-layer chromatography analysis

**Figure 5:**
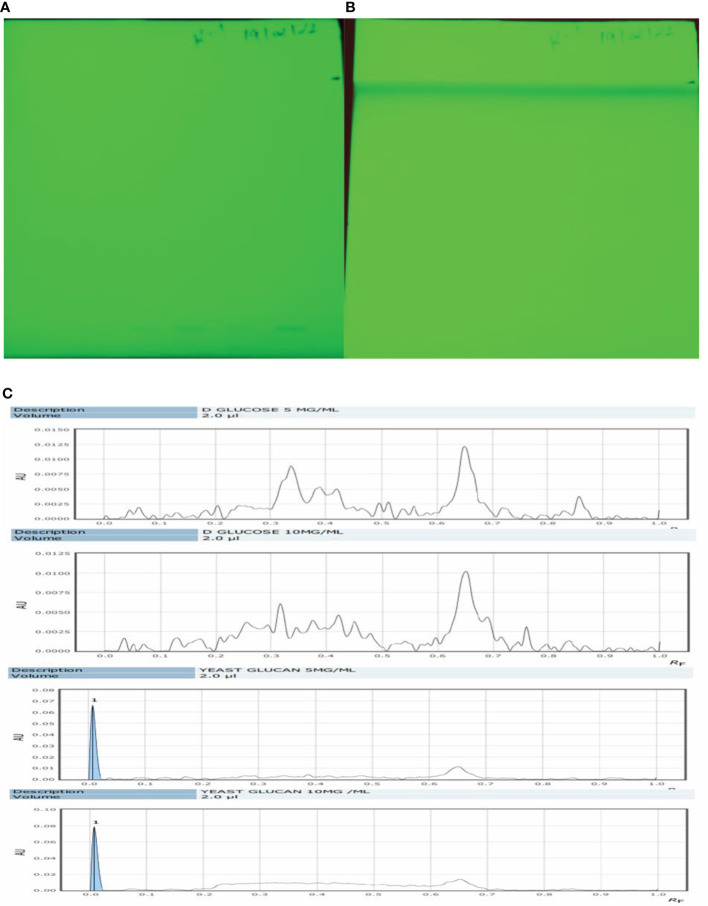
HPTLC analysis. **(A)** The silica plate after the spotting of the sample. **(B)** After movement in mobile phase N-butanol/methanol/water in the ratio 5.2:2.7:2.1 (v/v/v). **(C)** Recorded spectra in HPTLC. d-Glucose was taken as standard and showed its major peaks at Rf (retention factor) value of 0.6–0.7. The same pattern was observed in the case of β-glucan with peaks at 0.6–0.7. HPTLC, high-performance thin-layer chromatography.

Qualitative analysis of yeast β-glucan samples was performed using the CAMAG Application System (Muttenz, Switzerland). The developed plate was visualized at 254 nm using a Reprostar 3 Digital camera System (CAMAG) as shown in **Figures 5A, B**. d-Glucose was taken as a standard sample. The peaks of the yeast β-glucan sample were found similar to the peaks of d-glucose (**Figure 5C**). An analysis of carbohydrates showed that glucose content was present in yeast β-glucan.

### Particle size analysis of prepared β-glucan

**Figure 6:**
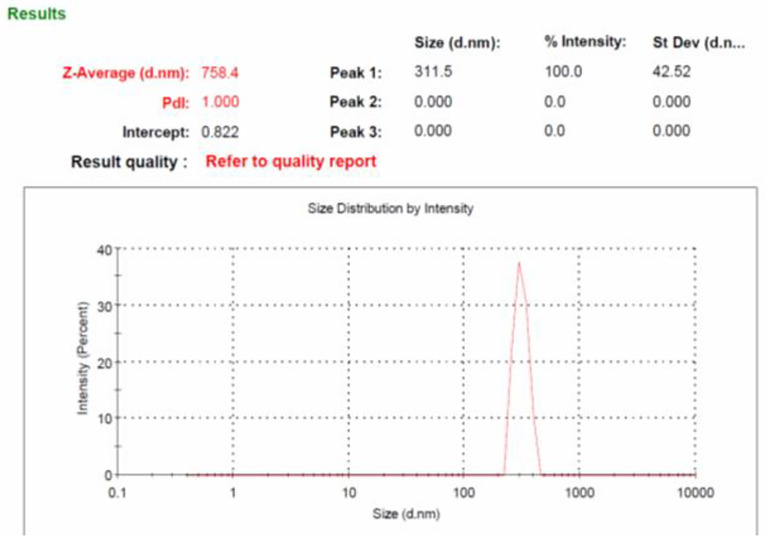
Particles size analysis of yeast β-glucan particles. The log-normal, volume-average particle size distribution of typical batch of spray-dried β-glucan powder (GP), as assessed by laser scattering.

Size of the β-glucan analyzed by Malvern Zeta sizer Ver. 7.03 was found on an average of 758.4 nm (**Figure 6**). The dispersant medium used was water. Particle size was determined by using a device (Mastersizer 2000, Malvern Instruments, Malvern, UK) as described previously (38) to determine if the cells are in the appropriate size range of 1–10 μm for the phagocytosis. According to Sharma et al. (38), 1–10-μm size of particles is favorable for uptake by cells. Moreover, they defined that particles ranging from 3 to 4 μm have efficient internalization properties (39). Briefly, about 5 mg of particles was dry-mixed with an equivalent amount of sodium lauryl sulfate (SLS) and suspended in 1 ml of Milli-Q water by vortex mixing. This slurry was added to the sampling beaker of the instrument until a laser obscuration factor of >10% was achieved. The average size (n = 3) in μm was determined for all formulations. Moreover, the polydispersity index (PdI) value that is used to measure the particle uniformity was found to be 1.00. PdI values reflect particle size distribution in which a large particle size shows a larger size distribution of particles, while a smaller PdI value indicates smaller size distribution of particles (40). Smaller PdI values of particle size analysis indicate higher homogeneity among particles.

### Scanning electron microscopy of β-glucan

**Figure 7:**
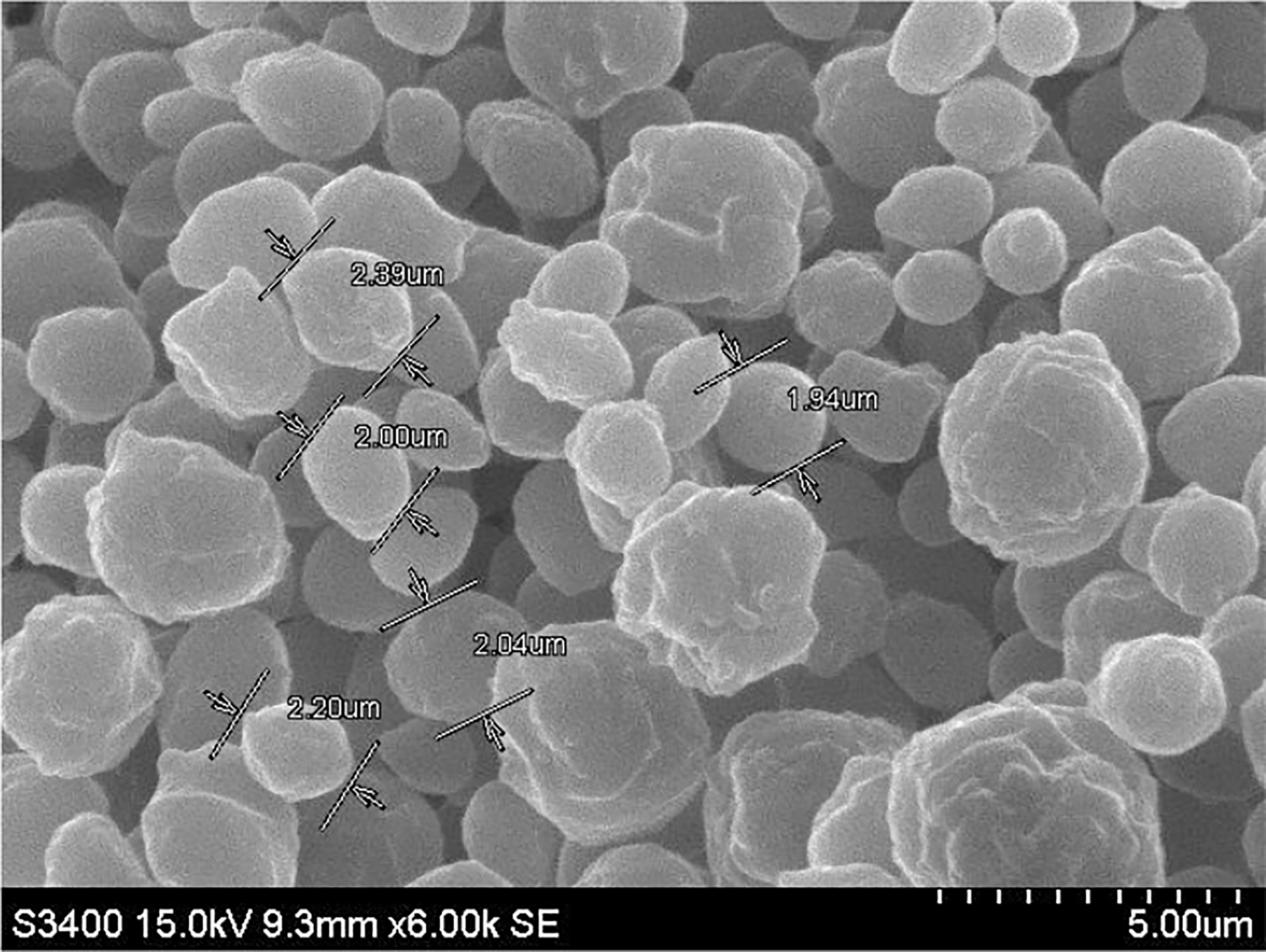
Morphological representation by scanning electron microscopy images of β-glucan extracted from *Saccharomyces cerevisiae* at 5-µm magnification.

Scanning electron microscopy of β-glucan extracted from *S. cerevisiae* was investigated on a 5-µm magnification scale. The shape of the extracted particles was spherical, and the particle size of the sample was about 1.94–2.39 µm, which was suitable for the uptake by cells as shown in **Figure 7**.

### Antioxidant activity by reducing power assay and DPPH assay

**Figure 8:**
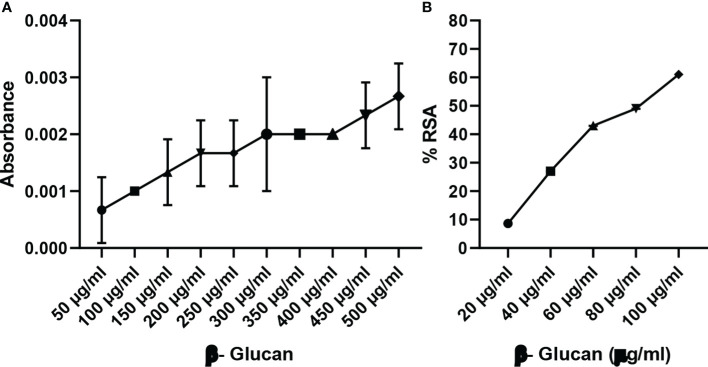
**(A)** Reducing power of yeast β-glucan. **(B)** DPPH assay. Free radical scavenging activity of yeast β-glucan was observed and was found to increase with increase in concentration. IC_50_ was found at 82 μg/ml. All the data are expressed as the mean ± standard deviation of the mean (SD) from triplicate experiments.

Reducing power is a colorimetric method of electron transfer to an oxidant from an antioxidant. It measures the reducing ability of the antioxidant from blue ferric tripyridyltriazine complex to its ferrous form. This results in a change of absorbance. In our study, maximum reducing power was measured at 500 μg/ml as shown in **Figure 8A**. Reducing power was measured according to absorbance, and absorbance was found to increase with the increase in concentration except for a few concentrations.

DPPH is used for the estimation of antioxidant activity. The reaction was examined for its ability to lower the DPPH solution’s absorbance at 517 nm. A rising trend in scavenging activity with the rise in concentration was observed by Divya et al. (31), the same as in our test; there was a continuous trend found with an increase in concentration (**Figure 8B**). All the data are expressed as the mean ± standard deviation of the mean (SD) from triplicate experiments (n = 3).

### Cell viability

**Figure 9:**
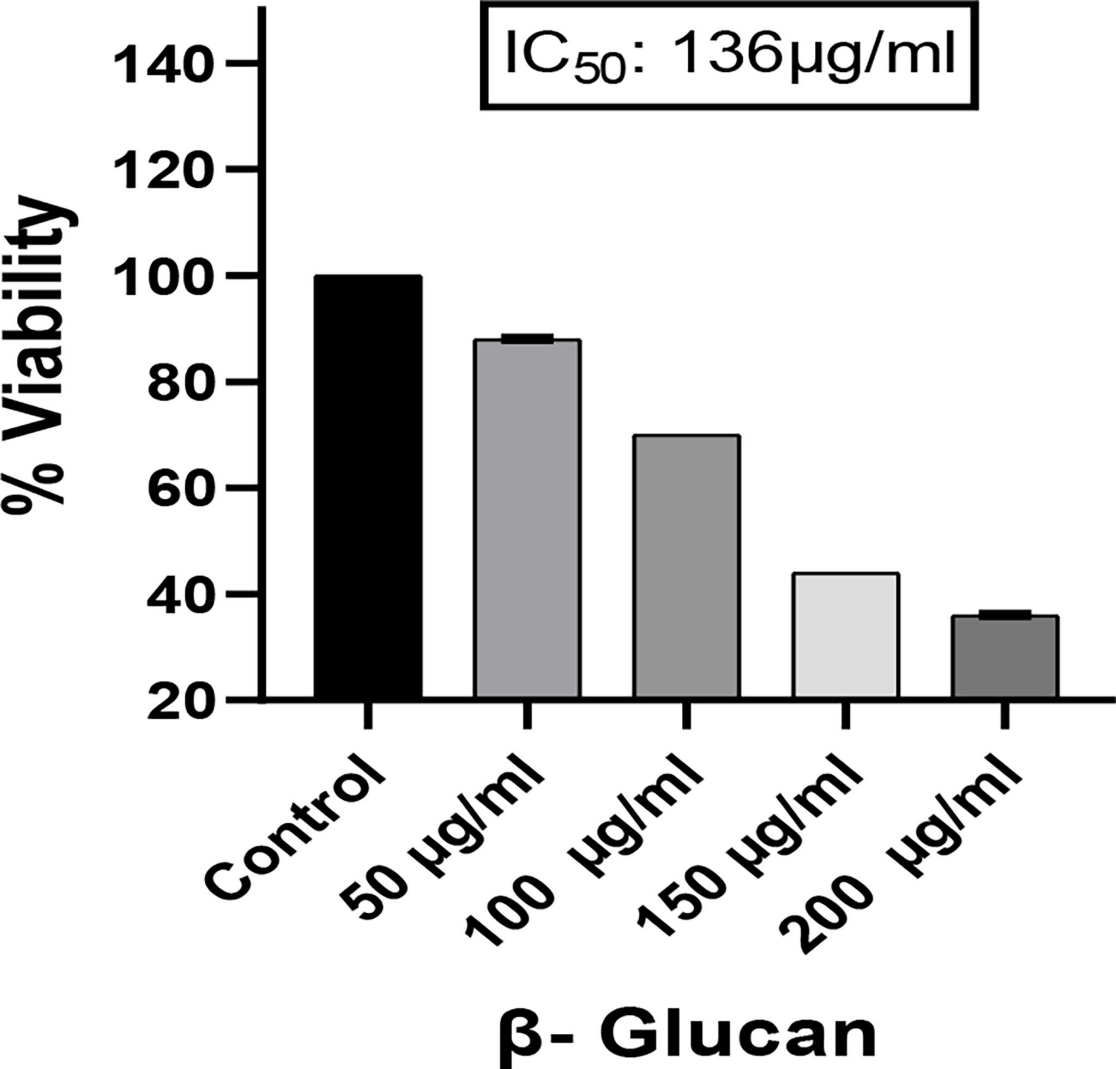
Effect of yeast β-glucan on cell viability of HeLa cell lines at 24 h using MTT assay. HeLa cell mortality was found to increase with increase in the concentration of treatment. A probability value of p < 0.05 was considered to be statistically significant. All the data are expressed as the mean ± standard deviation of the mean (SD) from triplicate experiments.

Yeast β-glucan potential was evaluated using an MTT assay. The proliferation of the cancer cell line HeLa was dramatically slowed after a 24-h treatment with various doses (50–200 μg/ml) of yeast β-glucan as shown in **Figure 9**. From these doses, the IC_50_ against the HeLa cells, which was found at 136 μg/ml and concentrations below and above IC_50_, were used for further experiments.

### Cellular reactive oxygen species generation upon yeast β-glucan exposure

**Figure 10:**
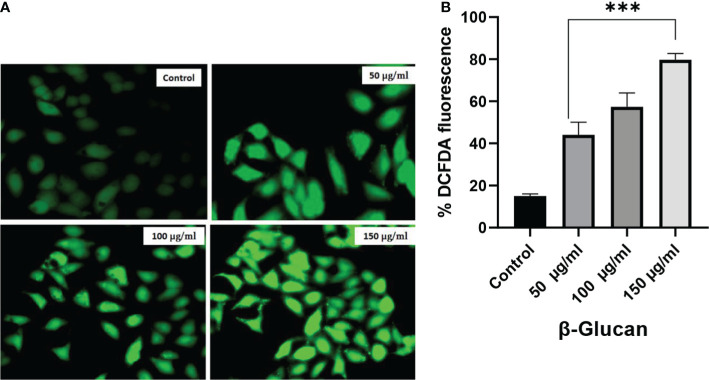
**(A)** Effect of yeast β-glucan on cellular ROS generation. Overproduction of cellular ROS in HeLa cells was induced by treatment with yeast β-glucan in 50–150 μg/ml for 24 h. Relative fluorescence of ROS was detected by cell membrane-permeable fluorescent dye H2DCFDA. Error bars are an indication of standard deviation. **(B)** One-way ANOVA performed with the help of GraphPad Prism 8.0 software showing 50, 100, and 150 μg/ml treatments as significant with p-value less than 0.05 (p < 0.05). Quantitative estimation performed with ImageJ software and fluorescence intensity measured as intensity of DCFDA. Minimum ROS generation in terms of DCFDA fluorescence was observed in control well, ROS generation increased with increase in treatment concentration, and maximum ROS generation was observed at 150 μg/ml. ROS, reactive oxygen species. ***Highly significant.

The production of ROS is important in the development of chronic disorders like cancer. Cellular ROS generation is a process of the living system for the maintenance of regulatory metabolism. In normal cells, ROS generation and elimination can be managed by a free radical scavenging system, but in the case of cancer cells, there is a high level of ROS produced to manage enhanced metabolic activities. This enhanced ROS generation can lead to the damage of genetic material, lipids, and proteins, which can ultimately lead to the apoptosis of cells. Chemotherapeutic compound generates high ROS resulting in the suppression of cancer. To determine the role of ROS generation in cancer cells, we have tested yeast β-glucan in HeLa cells from 50 to 150 μg/ml of concentration (**Figure 10A**). A significant ROS generation was observed with the increase in concentrations as shown in **Figure 10B**. One-way ANOVA with the help of GraphPad Prism 8.0 software showed a p-value less than 0.001 (p < 0.001), making these results highly significant.

### DAPI staining assay

**Figure 11:**
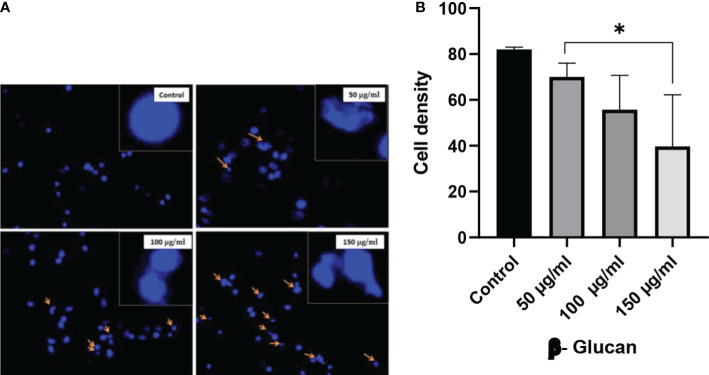
**(A)** Fluorescence microscopy images of HeLa cells stained with DAPI. In control well, nucleus was found intact and in regular shape, while in treatment wells, nucleus fragmentation and morphological changes were observed. These changes were increased with the increasing concentration. The arrows show chromatin and DNA fragmentation due to yeast β-glucan treatment. **(B)** One-way ANOVA performed with the help of GraphPad Prism 8.0 software showing all the treatments significant with p-value = 0.0276 (p < 0.05). Quantitative estimation was performed with ImageJ software. **less significant.

Apoptotic cells having condensed and fragmented DNA was observed under a fluorescence microscope with the help of DAPI staining as shown in **Figure 11**. HeLa cells were stained with DAPI after 24-h exposure to treatment ranging from 50 to 150 μg/ml of concentrations (**Figure 11A**). In this test, the nucleus of untreated cells was found intact, while treated cells were present with visible morphological changes. All the treatments were found to be significant in one-way ANOVA performed with the help of GraphPad Prism 8.0 software (**Figure 11B**) having a significant level at p < 0.05 and quantitative estimation performed with ImageJ software.

### LysoTracker staining

**Figure 12:**
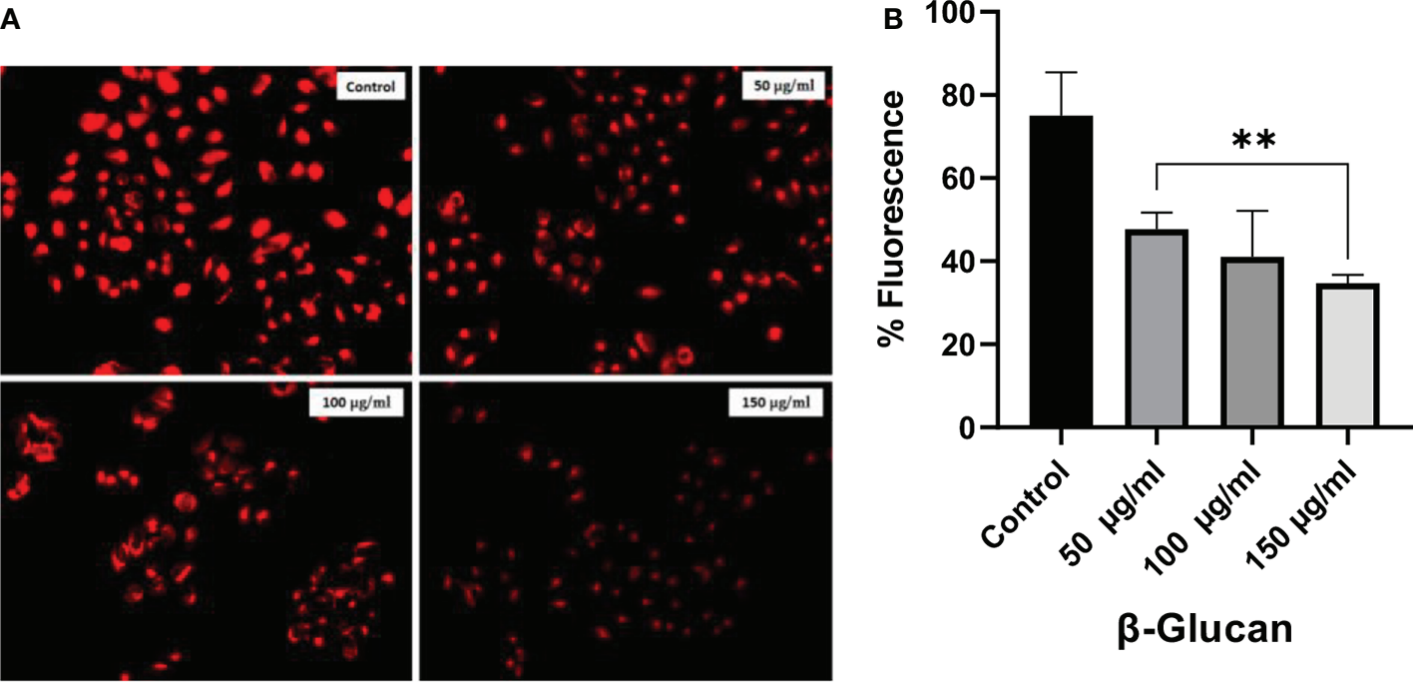
LysoTracker Red stained HeLa cells. **(A)** Red fluorescence intensities of the HeLa cells were significantly decreased after exposure to 50, 100, and 150 μg/ml of concentration showing the decrease in number of acidic organelles with the increase in treatment. **(B)** One-way ANOVA was performed with the help of GraphPad Prism 8.0 software using GraphPad Prism showing 50, 100, and 150 μg/ml as significant with a p-value = 0.0012 (p < 0.05). Quantitative estimation performed with ImageJ software. *Least significant.

LysoTracker is a dye used to detect the acidic organelles of the cells. LysoTracker staining fluorescence was found to decrease with an increase in concentration (**Figures 12A, B**) showing similarity with the results observed by the test by (41).

### MitoTracker staining

**Figure 13:**
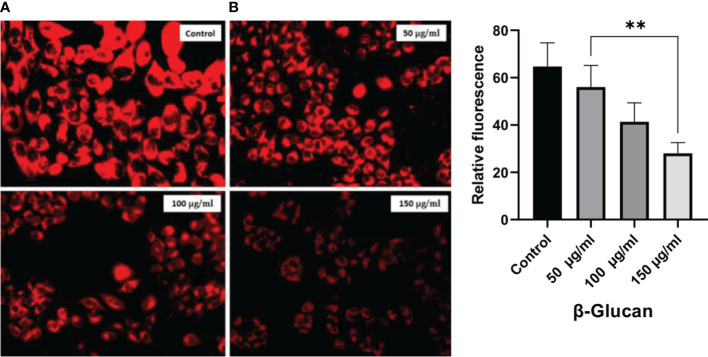
**(A)** Significant reduction in red fluorescence with an increase in treatment dose. **(B)** One-way ANOVA with the help of GraphPad Prism 8.0 software and quantitative estimation performed with ImageJ software showing significant results of p < 0.05 (p-value 0.0036). **less significant.

Mitochondria have several very crucial activities such as energy production and regulation of biosynthetic and metabolic pathways for the growth and survival of the cells. To measure the mitochondrial membrane potential, we observed the case of β-glucan-treated and untreated HeLa cells with the help of MitoTracker red staining dye. There was a significant reduction seen in fluorescence of treated HeLa cells as shown in **Figures 13A, B**, following a similar pattern of fluorescence reduction with an increase in the concentration of treatment observed by (42).

## Discussion

Anticancer properties of yeast β-glucan are reported in many studies due to their effects on innate and adaptive immune systems. The use of β-glucans in cancer prevention and therapy, particularly for cervical cancer, is a potential therapeutic alternative. We have extracted β-glucan with the alkaline and acidic methods of the extraction followed by spray drying and further anthrone test performed to quantify the sugar. Anthrone test confirmed the presence of polysaccharides in the extracted β-glucan sample. FTIR analysis confirmed the structure of yeast β-glucan having 1,3 glycosidic linkage with the presence of C–O stretch, CHOH stretch, C–H stretch, and OH stretch. HPTLC analysis confirmed the polysaccharide nature of β-glucan due to the same peaks as d-glucose, and the same was observed by (27). A similar chromatography pattern as of d-glucose was observed in the β-glucan sample. With the help of the HPTLC analysis, we confirmed the presence of d-glucose as the major polysaccharide fraction in extracted β-glucan. Congo red has the ability to bind with the helical structure of sugar, so we confirmed it by Congo red staining of yeast β-glucan. The observed absorbance spectra were showing similarities with the Congo red absorption spectra obtained by (26). It is reported that triple-helical β-1,3-1,6-d-glucan exhibits a bathochromic shift, while linear β-1,3-d-glucan does not exhibit a bathochromic shift. Hence, we confirmed that isolated yeast β-glucan has β-1,3-1,6 glycosidic linkage. A similar type of experimental data was also observed by (43) with the help of Congo red staining. Particle size analysis of isolated yeast β-glucan showed a PdI value of 1, which clearly indicates that particles have a broad size distribution, and for the confirmation of actual size, we performed scanning electron microscopy, which showed a size range between 1.94 and 2.43 µm and spherical shape of the particles.

Radical scavenging activity was evaluated by DPPH assay. Radical scavenging activity is the activity of an antioxidant that helps prevent damage caused by free radicals through the process of neutralizing free radicals by donating an electron to them. In a study by Divya et al. (2020) (31), the scavenging activity of β-glucan isolated from *Eleusine coracana* showed a rising trend when compared with control, while in our study, yeast β-glucan showed scavenging activity in all the concentrations with little variations with IC_50_ equal to 82 μg/ml. The impact of β-glucan on cell survival was assessed by an MTT assay to check cytotoxic potential. The rate of tetrazolium reduction is directly proportional to the survival and proliferation of the cells. MTT is endocytosed by cells, and after reduction by mitochondrial enzymes, it is transported to the surface of the cell to form formazan crystals. It is reported that endocytosis of the MTT is not responsible for cell death, while metabolism and exocytosis of MTT cause cellular damage (44). Mortality of the HeLa cells was found to increase with the increase in treatment concentration of β-glucan, and maximum cell death was found at a concentration of 200 μg/ml.

**Figure 14:**
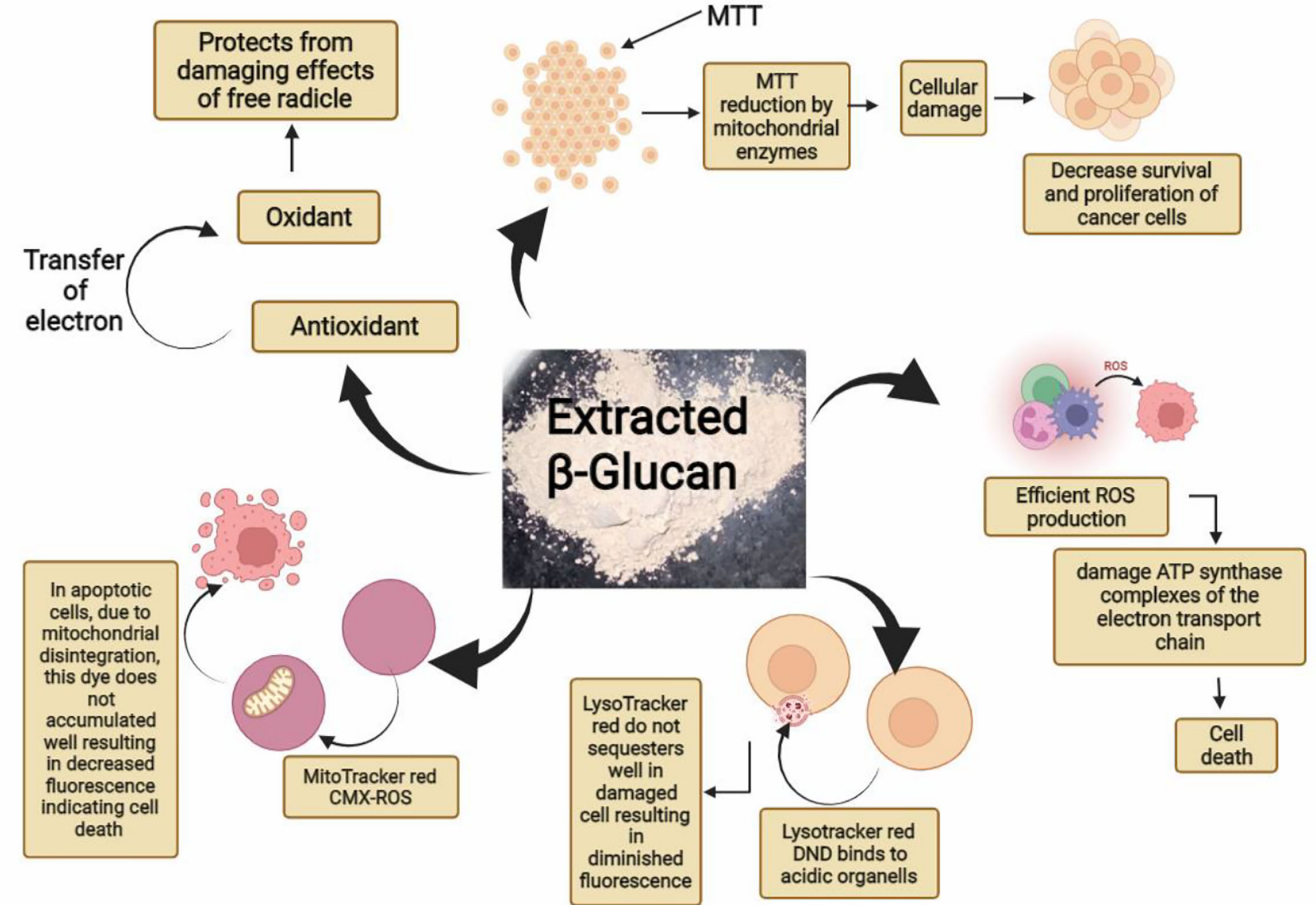
Graphical abstract showing various reported activities of β-glucan in our study.

ROS generation can result in cell apoptosis as observed during many studies. In our study, ROS generation and mitochondrial damage are frequently observed in cells exposed to yeast β-glucan treatment showing antitumor effects. The generation of excessive ROS can damage NADH dehydrogenase, cytochrome *c* oxidase, and ATP synthase complexes of the electron transport chain as shown in **Figure 14**. Damage to these complexes results in the destruction of mitochondria, ultimately shutting down the energy production of mitochondria (45). An excess amount of ROS generation results in an oxidative burst, which occurs due to an imbalance between the production and destruction of ROS. In normal physiological conditions, prooxidants and antioxidants remain in a balanced state to protect from damage by free radicals. Moreover, antioxidants can neutralize ROS by converting them into less reactive species (46), so in our study, oxidative bursts by ROS generation and antioxidant activity were found to have no relation. These two are independent activities shown by β-glucan. ROS generation was found to increase with the increase in treatment concentration of β-glucan, and this increased ROS production leads to damage to the mitochondria as previously reported, and it will further lead to cell death.

Staining with DAPI showed that yeast β-glucan has the potential to kill cancerous cells by DNA fragmentation. DAPI is a hydrophobic dye that preferentially binds to the AT region of the minor groove of the DNA. DAPI can permeabilize dead or fixed cells, and it can also stain living cells. Visible morphological changes were seen and indicated with arrows in our study. Morphological changes in the nucleus were found to increase with the increase in the concentration of yeast β-glucan. The numbers of acidic organelles were found to decrease with an increase in treatment concentration of β-glucan as observed with the help of LysoTracker staining. LysoTracker red DND-99 dye enters the cell *via* diffusion and sequesters in lysosomes of viable cells due to their acidic pH, leading to an intense red fluorescence. In apoptotic cells, due to a change in pH (increased pH), LysoTracker red does not sequester well, resulting in diminished fluorescence (47). The same decreased fluorescence was observed in our study, indicating the apoptosis of the HeLa cells by the treatment of yeast β-glucan.

MitoTracker Red CMX-ROS is a cationic dye and accumulates in the mitochondrial matrix due to the negative mitochondrial membrane potential. In apoptotic cells, due to mitochondrial disintegration, this dye does not accumulate well, resulting in decreased fluorescence (48). Mitochondrial membrane potential was found to significantly decrease with the increasing concentrations of yeast β-glucan, showing the similarity with the test performed by Pandey et al. (2021) (42), indicating the apoptosis of HeLa cells. Altogether, the findings of our study were in agreement that yeast β-glucan has the potential to decrease the growth and proliferation of HeLa cells, and further clinical trials can be carried out to use yeast β-glucan as a therapeutic for cervical cancer.

## Conclusion

β-Glucan is a known immuno-modulatory agent having an impact on a wide range of cells including T cells, natural killer cells, antigen-presenting cells, and macrophages, thereby affecting both the innate and adaptive immune systems. Our findings reveal that yeast β-glucan has good antioxidant and anticancer properties and has the potential to suppress the HeLa cancer cell proliferation. β-Glucan has the ability to generate ROS in HeLa cells with the help of various inflammatory pathways and oxidative bursts, thereby leading to the apoptosis of cancer cells, so it should be further investigated to determine its complete antitumor potential. By the analysis of mitochondrial membrane potential and LysoTracker staining, it was observed that β-glucan has the ability to induce apoptosis in HeLa cervical cancer cells by the modulation of the immune system. Hence, we concluded that β-glucan leads to apoptosis of cervical cancer cells, and for this purpose, further research and clinical trials should be carried out.

## Data availability statement

The raw data supporting the conclusions of this article will be made available by the authors, without undue reservation.

## Author contributions

MS. and DY conceived and designed the project, collected the data from the literature and databases, and wrote the manuscript. TU, RT, MS, and DY performed the experiments and analyzed the data. FK, LA-K, PP, AS, NA, and NMA were involved in editing and writing the manuscript. DY and MS provided the lab facilities. All authors contributed to the interpretation and discussion of the results. All authors have read and approved the final version of the manuscript.

## Funding

Princess Nourah Bint Abdulrahman University researcher, supporting program number (PNURSP 2022 R82) Princess Nourah bint Abdulrahman University, Riyadh Saudi Arabia.

## Conflict of interest

The authors declare that the research was conducted in the absence of any commercial or financial relationships that could be construed as a potential conflict of interest.

## Publisher’s note

All claims expressed in this article are solely those of the authors and do not necessarily represent those of their affiliated organizations, or those of the publisher, the editors and the reviewers. Any product that may be evaluated in this article, or claim that may be made by its manufacturer, is not guaranteed or endorsed by the publisher.
